# Profiling of 1-aminocyclopropane-1-carboxylic acid and selected phytohormones in *Arabidopsis* using liquid chromatography-tandem mass spectrometry

**DOI:** 10.1186/s13007-024-01165-8

**Published:** 2024-03-16

**Authors:** Michal Karady, Pavel Hladík, Kateřina Cermanová, Petra Jiroutová, Ioanna Antoniadi, Rubén Casanova-Sáez, Karin Ljung, Ondřej Novák

**Affiliations:** 1 Laboratory of Growth Regulators, Institute of Experimental Botany, Palacký University, The Czech Academy of Sciences & Faculty of Science, Olomouc, CZ-783 71 Czechia; 2 Department of Forest Genetics and Plant Physiology, Umeå Plant Science Centre (UPSC), Swedish University of Agricultural Sciences, Umeå, SE-901 83 Sweden; 3 Department of Plant Physiology, Umeå Plant Science Centre (UPSC), Umeå University, Umeå, SE-901 87 Sweden

**Keywords:** Ethylene, 1-aminocyclopropane-1-carboxylic acid, ACC, Plant hormones, Auxin, Cytokinin, Abscisic acid, Jasmonic acid, Salicylic acid, Mass spectrometry, Liquid chromatography, Arabidopsis

## Abstract

**Background:**

Gaseous phytohormone ethylene levels are directly influenced by the production of its immediate non-volatile precursor 1-aminocyclopropane-1-carboxylic acid (ACC). Owing to the strongly acidic character of the ACC molecule, its quantification has been difficult to perform. Here, we present a simple and straightforward validated method for accurate quantification of not only ACC levels, but also major members of other important phytohormonal classes – auxins, cytokinins, jasmonic acid, abscisic acid and salicylic acid from the same biological sample.

**Results:**

The presented technique facilitates the analysis of 15 compounds by liquid chromatography coupled with tandem mass spectrometry. It was optimized and validated for 10 mg of fresh weight plant material. The extraction procedure is composed of a minimal amount of necessary steps. Accuracy and precision were the basis for evaluating the method, together with process efficiency, recovery and matrix effects as validation parameters. The examined compounds comprise important groups of phytohormones, their active forms and some of their metabolites, including six cytokinins, four auxins, two jasmonates, abscisic acid, salicylic acid and 1-aminocyclopropane-1-carboxylic acid. The resulting method was used to examine their contents in selected *Arabidopsis thaliana* mutant lines.

**Conclusion:**

This profiling method enables a very straightforward approach for indirect ethylene study and explores how it interacts, based on content levels, with other phytohormonal groups in plants.

**Supplementary Information:**

The online version contains supplementary material available at 10.1186/s13007-024-01165-8.

## Background

Plant hormones represent a chemically very diverse group of bioactive compounds, affecting practically all stages of plant ontogenesis. One of the currently established classes of phytohormones is based on a simple two-carbon molecule ethylene. Being a unique gaseous plant hormone, it controls many processes affecting plant growth and is involved in important traits regulation like fruit ripening, seed germination, abscission, senescence, flooding responses etc [1]. Ethylene production in seed plants originates from Yang cycle and starts by a conversion of the sulfuric amino acid methionine to S-adenosyl-L-methionine (SAM), mediated by SAM-synthetase. Then, the enzyme ACC synthase (ACS) forms 1-aminocyclopropane1-carboxylic acid (ACC) and 5’-methylthioadenosine (MTA) from SAM. ACC undergoes oxidation by the enzyme ACC oxidase (ACO), resulting in ethylene and byproducts. Ethylene can move locally by diffusion in certain plant tissues or between plants, serving both as short-range and long-distance communication [2], or its relocation and subsequent production can be realized indirectly, through the transport of ACC, its direct biochemical precursor in seed plants [3]. ACC is a simple, non-proteinogenic amino acid discovered in 1979 [4]. It can undergo conjugation, is a subject for intrinsic transport mechanism, or it can be metabolized by rhizosphere bacteria [5]. Seed plants can absorb and transform ACC into ethylene very well. This allows researchers to use ACC treatment as a replacement for ethylene gas to trigger ethylene-related reactions. This approach led to discoveries of novel ACC responses, which are distinguishable and decoupled from possible ethylene effects, thus pointing towards ACC as a novel signaling molecule [6]. Auxins (AUX), with the prominent bioactive molecule indole-3-acetic acid (IAA), represent perhaps the most important plant hormone group and IAA is one of the most studied plant compounds, greatly affecting all stages of plant development [7]. IAA metabolism is complex, with indole-3-acetyl glutamate (IAA-Glu) and indole-3-acetyl aspartate (IAA-Asp) being the main conjugation products, whereas 2-oxindole-3-acetic acid (oxIAA) is one of the important catabolic metabolites [8]. Many facets of auxin and ethylene interplay have been uncovered and a strong link between these two phytohormones exists [9]. Cytokinins (CK) are adenine derivatives with isoprene-derived side chain at the *N*^6^-terminus and a class of plant hormones involved in cell division, apical dominance, leaf senescence, stress responses etc., with isopentenyladenine (iP), *trans-* and *cis*-zeatin (*t*Z, *c*Z) as bioactive molecules. Their respective ribosides are iPR, *t*ZR and *c*ZR, with *t*ZR recently shown to be involved in a very significant role for long-distance signaling of crucial nitrogen availability [10, 11]. Abscisic acid (ABA), salicylic acid (SA), jasmonic acid (JA) and its bioactive isoleucine conjugate form (JA-Ile) are often described as stress phytohormones, as they are mainly involved in biotic and abiotic stress responses. However, their activities stretch to many other aspects of plant ontogenesis [12–16].

All of the mentioned phytohormones usually exhibit multiple effects at the same developmental stage, or, in contrast, many hormones can affect a single process simultaneously. Their specific activity depends on a plethora of various factors, concentration being one of the most important. Knowing how to assess their exact quantity is therefore crucial in elucidating their mode of action and related consequences for plant existence. The levels of plant hormones can change greatly depending on the type of plant tissue, with concentrations being very or extremely low for most of them [17]. A method for the accurate measurement of these compounds should therefore be able to encompass all the varying concentrations while, at the same time, overcoming many limitations usually caused by the very complex plant tissue.

The most common detection method for ethylene, as a volatile compound, is gas chromatography (GC). An alternative is provided by specialized detectors, which usually lack the broad applicability of GC, however they offer better sensitivity or improve some other important aspect of ethylene detection [18]. Since its discovery, ACC content has been determined mostly by indirect GC measurement through liberation of ethylene from ACC [19, 20].

Liquid chromatography (LC), coupled with tandem mass spectrometry as a detector (LC-MS/MS), is in general the most prominent analytical method for small molecules quantification from complex matrices and is prevalent in plant hormone profiling, including auxins, cytokinins, abscisic acid, jasmonates and salicylic acid [21, 22]. LC with other detectors has been used for direct ACC quantification [23–26] with some more recent methods employing LC-MS/MS [27–30], with many other examples in literature.

Several strategies exist for plant material preparation to make it suitable for LC-MS/MS analysis. Usually, the plant is extracted using a mixture of polar and nonpolar solvents, followed by greatly varying processing steps. These most often include sample purification performed by means of solid/liquid-liquid phase extraction (SPE, LLE) or other purification steps for interfering compounds removal. Different approaches may include, for example, derivatization, for specific enhancement of target compound properties. Due to the extraordinarily diverse chemical nature of phytohormones, the availability and testing of these protocols is a decisive factor in their analysis and detection [22], nevertheless, the inclusion of most phytohormones and their metabolites in one method is very much possible [31, 32].

As ACC is a direct precursor to one of the classic phytohormones and might be a new signaling molecule in plants, availability of validated quantification method is essential. Similar importance should be placed on simultaneous quantification of other plant hormones with ACC, as these almost never act alone and their interplay with ethylene pathway has been shown many times [33, 34]. This remains the main aim of this work, where we present a simple purification method of a minute sample amount with subsequent LC-MS/MS analysis for quantification of fifteen phytohormonal compounds, consisting of ACC and the mentioned plant hormones or their metabolites.

## Results and discussion

### LC-MS/MS method development and optimization

Due to broad applicability, the C18 columns are preferred for most type of chromatographic analyses, with wide availability of instruments [35], and therefore they are usually the first choice in LC-MS/MS method development. For the subsequent step, selection of LC mobile phase should reflect the need for its volatility, and for its ability to retain our particularly acidic target compounds as ACC. We have tested two separation columns, taking in account the high polarity and thus a predictable poor retention of ACC. The selected C18 columns for testing, Kinetex Biphenyl (100 × 2.1 mm, 1.7 μm, Phenomenex) and Kinetex Polar (150 × 2.1 mm, 2.6 μm, Phenomenex), both provide new core-shell based particles with 100% aqueous stability, targeting enhanced polar compounds retention and in case of Biphenyl column, providing also orthogonal selectivity. For both columns, ACC standard was eluted at the very beginning of the gradient, which is usually unsuitable for analysis from complex plant matrices [36]. This problem is further exacerbated by low molecular weight of ACC, which can lead to low sensitivity and unwanted immersion in background noise. To mitigate this effect, we decided to employ a derivatization approach for ACC quantification. As it is essentially an amino acid, there were many derivatization methods readily available [37] and derivatization has been used for ACC analysis previously [25, 27, 38–40]. We opted for 6-aminoquinolyl-N-hydroxysuccinimidyl carbamate derivatization in the form of AccQ-Tag Ultra Derivatization Kit from Waters, as it comes in commercially available pre-mix, requires minimal amount of work and provides a very fast (< 10 min) and tested reaction, producing stable amino acid derivatives with fragmentation suited for tandem mass spectrometry approach [41–43]. In some cases, this derivatization was already used for ACC, however with non-validated methods [23, 30, 44, 45]. None of the other phytohormones and related compounds presented in this work are capable of being derivatized with this approach, most likely as they do not have an amino- group suitable for the reaction [41]. The proposed derivatization reaction of ACC is displayed in Additional file 1: Figure S1. When auxins were prepared and injected in derivatization solution, they were either not observed, or their chromatography was severely diminished. This is most likely due to very basic pH of AccQ-Tag solution (pH ~ 8–9), as this pH is needed for the derivatization reaction to occur [41]. The samples and standards were therefore dissolved in two different solutions – 10% acetonitrile (ACN) (v/v) for all compounds, except ACC, which was derivatized after evaporation. Every sample resulting from this method needs therefore to be analyzed twice, as derivatized for ACC and as non-derivatized for the remaining compounds, with the optimized gradient.

**Fig. 1:**
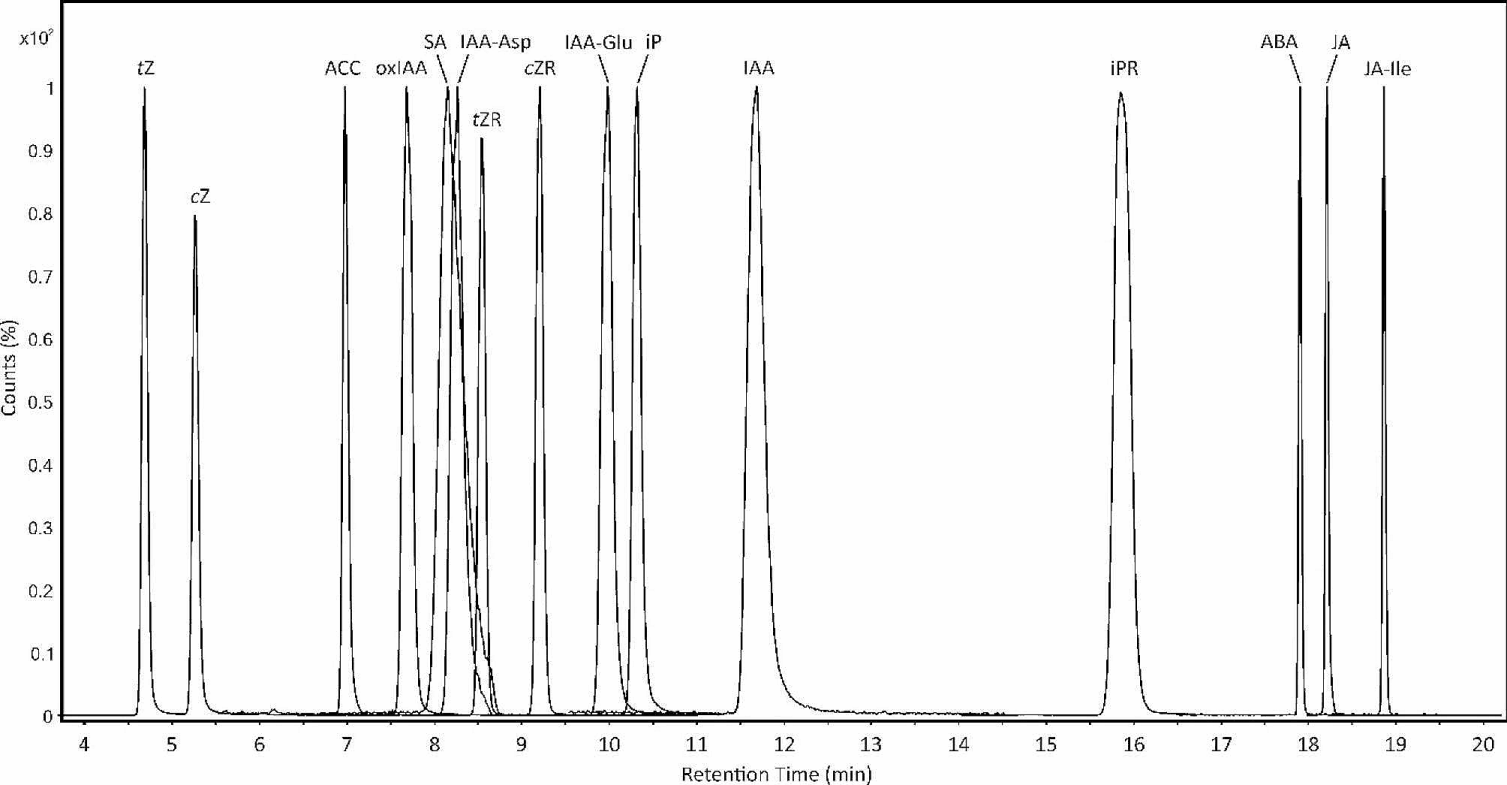
Optimized LC-MS/MS chromatogram of examined analytes. Separation of analyte standards using a Kinetex Polar (150 × 2.1 mm, 2.6 μm) column and gradient elution with 0.03% formic acid and acetonitrile as mobile phase A and B, respectively. The gradient used is 23 min long and its composition was 0 min − 99% A, 8 min – 91.5% A, 9 min – 90% A, 15 min – 88% A, 20 min – 2% A, 21 min – 99% A, 23 min – 99% A. Measurements were performed on Agilent 6495B Triple-Quad LC/MS coupled to 1260 Infinity II LC system

Standard working solutions of investigated compounds and their respective internal standards (IS; for *c*ZR [^2^H_5_]-*t*ZR was used as IS) were injected into LC-MS/MS system. As mentioned, they were dissolved in 10% ACN (v/v) or evaporated and derivatized (ACC; internal standard [^2^H_4_]-ACC). Their retention time together with precursor ion mass was monitored in Full Scan mode, with further fragment ion identification in Product Ion mode. Collision energy, ion source parameters and polarity were then optimized for each compound individually and the results used to construct the final gradient. The optimized mass spectrometry and detection parameters for all compounds, including IS, are shown in Table 1. The observed precursor mass and fragmentation values for ACC and [^2^H_4_]-ACC correspond to the expected derivatization pattern (see Additional file 1: Figure S1), with fragmentation resulting in the predicted amino-quinoline moiety having a *m/z* (mass-to-charge) of 171.0 [41]. Derivatization of ACC and its internal standard resulted in much more suitable retention time and provided very good repeatability and detection.

The chromatography with peaks of the monitored phytohormones, resulting from the optimized gradient, is shown in Fig. 1. The elution order of peaks for the separated compound groups (CK, JA, SA, ABA, AUX) corresponds with orders observed on C18 columns before [46, 47], with the exception of oxIAA and IAA-Asp.

**Table 1:** Optimized LC-MS/MS parameters for analyzed compounds and their corresponding internal standards

Analyte	Retention time (minutes)	Precursor ion (m/z)	Fragment ion (m/z)	ESI Ionization mode (+/-)	Collison energy (eV)	Linear range (pmol)	R^2^	Internal standard
ACC	6.96	272.1	171.0	[M + H]^+^	18	0.00045–4.5	0.9995	[^2^H_4_]-ACC
[^2^H_4_]-ACC	6.89	276.1	171.0	[M + H]^+^	18	-	-	
*t*Z	4.67	220.2	136.1	[M + H]^+^	19	0.00036–0.36	0.9996	[^13^C_5_]-*t*Z
*c*Z	5.24	220.2	136.1	[M + H]^+^	19	0.00036–0.36	0.9994	[^13^C_5_]-*c*Z
iP	10.3	204.2	136.0	[M + H]^+^	12	0.001–1	0.9982	[^2^H_6_]-iP
*t*ZR	8.57	352.1	220.1	[M + H]^+^	20	0.0001–0.5	0.9991	[^2^H_5_]-*t*ZR
*c*ZR	9.19	352.1	220.1	[M + H]^+^	20	0.001–1	0.9988	[^2^H_5_]-*t*ZR
iPR	15.85	336.1	204.1	[M + H]^+^	20	0.001–1	0.9997	[^2^H_6_]-iPR
[^13^C_5_]-*t*Z	4.66	225.1	141.1	[M + H]^+^	20	-	-	-
[^13^C_5_]-*c*Z	5.23	225.1	141.1	[M + H]^+^	20	-	-	-
[^2^H_6_]-iP	10.16	210.0	137.0	[M + H]^+^	12	-	-	-
[^2^H_5_]-*t*ZR	8.45	357.1	225.1	[M + H]^+^	20	-	-	-
[^2^H_6_]-iPR	15.62	342.1	210.1	[M + H]^+^	20	-	-	-
IAA	11.65	176.1	130.1	[M + H]^+^	24	0.018–36	0.9991	[^13^C_6_]-IAA
oxIAA	7.68	192.1	146.0	[M + H]^+^	12	0.009–90	0.9993	[^13^C_6_]-oxIAA
IAA-Glu	9.98	305.2	130.1	[M + H]^+^	24	0.09–90	0.9990	[^13^C_6_]-IAA-Glu
IAA-Asp	8.25	291.1	130.1	[M + H]^+^	36	0.0045–90	0.9995	[^13^C_6_]-IAA-Asp
[^13^C_6_]-IAA	11.64	182.1	136.0	[M + H]^+^	24	-	-	-
[^13^C_6_]-oxIAA	7.67	198.1	152.1	[M + H]^+^	12	-	-	-
[^13^C_6_]-IAA-Glu	9.96	311.2	136.1	[M + H]^+^	24	-	-	-
[^13^C_6_]-IAA-Asp	8.22	297.1	136.0	[M + H]^+^	36	-	-	-
ABA	17.89	263.2	153.1	[M + H]^−^	8	0.0018–3.6	0.9992	[^2^H_6_]-ABA
JA	18.19	209.2	58.8	[M + H]^−^	8	0.0018–18	0.9996	[^2^H_6_]-JA
JA-Ile	18.85	324.3	151.2	[M + H]^+^	16	0.0018–3.6	0.9986	[^2^H_2_]-JA-Ile
SA	8.14	137.1	92.8	[M + H^−^]	16	0.045–45	0.9988	[^2^H_4_]-SA
[^2^H_6_]-ABA	17.87	269.2	159.1	[M + H]^−^	8	-	-	-
[^2^H_6_]-JA	18.17	215.2	58.8	[M + H]^−^	8	-	-	-
[^2^H_2_]-JA-Ile	18.84	326.3	151.2	[M + H]^+^	16	-	-	-
[^2^H_4_]-SA	8.03	141.1	96.8	[M + H]^−^	16	-	-	-

*m/z* – mass-to-charge ratio, ESI – electrospray ionization, +/- – positive/negative, R^2^ – coefficient of determination, eV – electron volts. Linear range and R^2^ was not calculated for internal standards

These two compounds display a switched elution order, compared to previously published chromatographs separating auxin and related compounds on similar chromatographic columns [48, 49], most likely due to usage of different organic mobile phase (ACN vs. methanol, formic acid vs. acetic acid) and due to different column solid phase.

Final composition of mobile phase was 0.03% formic acid in deionized water (dH_2_O) (v/v) as phase A, and pure acetonitrile as phase B. The presence of formic acid at higher concentration was detrimental for auxins peak area and affected isomeric CK separation, whereas using acetonitrile instead of methanol as organic solvent proved to be also important for isomeric compounds separation and lowering the backpressure of the system. Similar effects of formic acid have been observed before [50]. The Biphenyl column had much higher backpressure, operating close to the maximum of our LC system (600 bar), whereas the Polar column proved to be effective for all compounds separation, with optimized gradient. The coefficient of determination (R^2^) showed reasonable linearity as it was very close to or higher as 0.999 for all observed and prepared calibration curves.

### Extraction of various plant hormones and related compounds from plant material

LC-MS/MS, with LC (often denoted as high-performance LC (HPLC) or ultra HPLC (UHPLC), depending on the requirements for operating pressure) became the main method for accurate phytohormone profiling [51]. Rapid advances in mass spectrometry instrumentation result mainly in enhanced sensitivity, resolution and mass accuracy, thus allowing for a low starting amount of fresh weight (FW) plant material (5-100 mg), enabling the use of internal standards and an ability to perform separation of many chemically different compounds in one run [32, 52]. The selection of extraction solution was based on previous works and some necessary precautions [47, 52–54], focusing on solvent ability to preserve the metabolic state of collected sample. Additionally, enzymatic activity is in most extraction cases the main culprit to address, for prevention of possible rapid metabolites turnover. This can be achieved mainly by applying cold or heated organic extraction solvent, with acid or base. This quenching is necessary for producing an extract, which would accurately reflex the amount of metabolites in plants or other specimen [55]. As first step, we opted for low temperature for all of the used tools, lab material and solvents throughout all performed extraction steps, aiming to keep their temperature around 3-4^o^C. The low temperature should help prevent enzymatic activity and degradation, however, it needs to be kept above freezing point to allow the solvents to remain in liquid state [56]. For extraction solvent, 10% acetonitrile (v/v dH_2_O) was chosen, with addition of 0.5% formic acid (v/v) to further prevent enzymatic activity. Corresponding IS were used for all of the monitored analytes, as they present an optimal way to account for losses during all of the steps of plant material extraction and profiling [17] and therefore provide a very accurate and unmatched tracing of analyte levels.

Sample preparation, which consists mostly of plant material extraction and purification, is still a major cornerstone of analysis and it can take up most of the time dedicated for whole quantification procedure, as plant tissue is one of the most challenging matrices [57]. We tried an approach employing only irreplaceable steps involved in the whole extraction process. These consists of adding 500 µL of extraction solution with mixture of IS and zirconium oxide beads to the frozen plant tissue, followed by tissue homogenization, centrifugation at 30 000 g, collection of the resulting supernatant (split in 250 µL for phytohormones, and 150 µL for derivatization and ACC analysis) and its evaporation to dryness. This was followed by reconstitution of the sample in 30 µL of 10% ACN (v/v) or in 18 µL of derivatization solution mixture (14 µL of borate buffer and 4 µL of derivatization powder, dissolved in ACN according to manufacturer’s instructions (see “Material and methods” section)). Evaporation and reconstitution, preferably in solution closely resembling the starting composition of mobile phase gradient, are crucial for concentrating the sample [58]. Similarity of starting mobile phase could not be achieved for the derivatized sample, however any incoherencies in peak shape or other parameters for ACC analysis were not observed. We would then try to inject the samples obtained in this way and quantify the phytohormones, using the optimized LC-MS/MS method. If any problems would have been observed for this initial approach, we would reconsider the previous extraction steps, choice of solutions etc. This type of analysis, not employing a SPE, LLE or other dedicated purification step, is now commonly used in other tissues or fluids analysis [59], however is still quite rare in small molecule profiling from plants. Finally, we were able to observe and quantify all of 15 analytes from 10 mg FW of *Arabidopsis thaliana* Col-0 plants harvested 10 days after germination (DAG).

### Method validation

Our method validation was based on assessing accuracy, precision, process efficiency (PE), matrix effect (ME) and recovery according to Matuszewski et al. [60], employing the postextraction addition. The results presented in Table 2 were acquired from four replicates. Accuracy was estimated by spiking 10 mg of 10 DAG old *A. thaliana* plants with a constant amount of IS and known, differing amounts of nonlabeled standards at the beginning of extraction, or at the end, before evaporation. The endogenous levels were subtracted from the measured analyte quantities in samples and the resulting values were compared with the known spiked amounts. The difference between them is expressed in Table 2 as a nominal level percentage (bias). These values stay on average well below 15%. Precision was expressed as % of relative standard deviations (RSD) and was below 5% for all examined analytes. Recovery (RE) is presented as a percentage of compounds retained for analysis after extraction steps.

**Table 2:** Method validation parameter summary (for detailed description see „method Validation“chapter)

Analyte	Spiked content (pmol)	Average Accuracy (%bias)	Average Precision (%RSD)	Average Process Efficiency (%)	Average Matrix Effect (%)	Average Recovery (%)
ACC	2-200	5.59	3.2	21.01	23.74	88.17
*t*Z	0.01–0.1	10.02	1.81	44.92	58.56	90.92
*c*Z	0.01-1	8.92	0.84	42.66	63.98	68.18
iP	0.01-1	6.12	2.71	39.12	49.19	80.4
*t*ZR	0.01-1	12.3	2.44	39.76	45.74	87.53
*c*ZR	1	12.16	2.05	62.43	75.53	82.65
iPR	0.01-1	12.65	1.94	56.12	83.94	65.1
IAA	0.1–100	12.72	1.63	50.38	80.08	63.53
oxIAA	10–100	11.81	1.82	44.93	59.88	75.17
IAA-Glu	0.1–100	8.63	3.2	77.57	95.95	81.71
IAA-Asp	0.1–100	7.48	2.06	66.13	81.82	80.95
ABA	0.1–100	10.39	1.62	72.15	106.27	69.85
JA	0.1–100	8.31	2.48	68.21	96.72	70.76
JA-Ile	1	6.98	4.95	17.69	48.12	36.74
SA	0.5–50	9.31	1.87	106.88	133.62	79.94

Values for Average Process Efficiency, Average Matrix Effect and Average Recovery are means ± SD; *n* = 4. Accuracy and precision were estimated by spiking 10 mg of FW *A. thaliana* with authentic standards (*n* = 4)

**Fig. 2:**
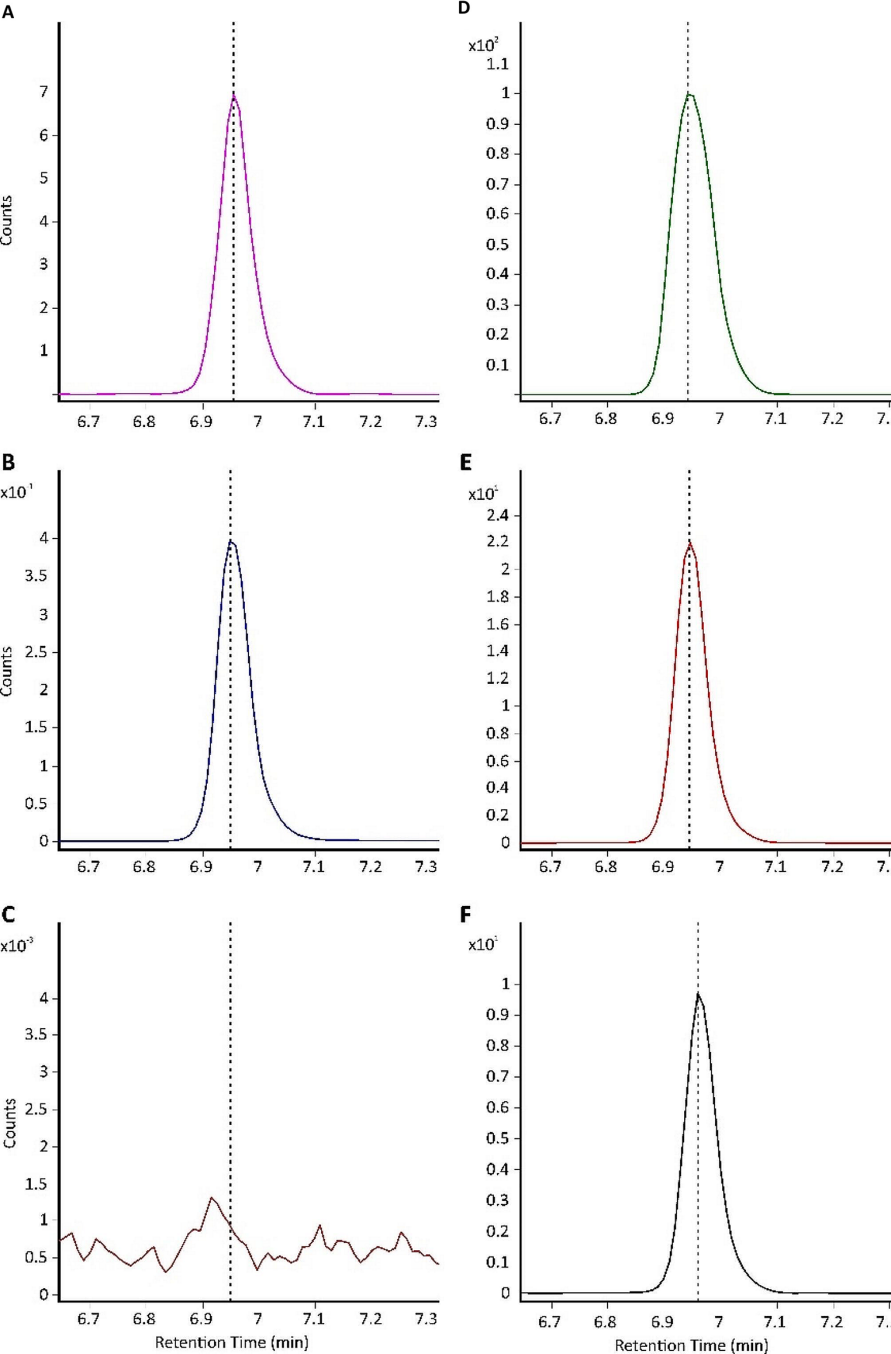
Representative MRM (multiple reaction monitoring) chromatograms of ACC standards and in vivo ACC. (**A**) - ACC peak from non-spiked *A. thaliana*; (**B**) - Peak of 10 fmol ACC standard; (**C**) - derivatization solution without ACC; (**D**) - ACC peak from *A. thaliana* spiked with 200 pmol ACC; (**E**) - ACC peak from *A. thaliana* spiked with 20 pmol ACC; (**F**) - ACC peak from *A. thaliana* spiked with 2 pmol ACC. *A. thaliana* amount was 10 mg FW for each spiking

We found the lowest values for JA-Ile (36.74%) and highest for *t*Z, its riboside and ACC (~ 87–90%). Matrix effect is an important parameter of method evaluation, as it can directly influence the response of mass detector and therefore directly interfere with accuracy and precision values. ME is essentially a change in the ionization efficiency of monitored analyte due to the occurrence of a coeluting compound, however there are many mechanisms, by which the plant matrix is believed to shape the analysis. Of these, the most important seems to be the competition for a charge between molecules and the resulting ionization efficiency or change of the sample viscosity affecting the droplet evaporation in the ion source. These effects however do not exert only detrimental, negative effects, for some analytes, they can lead to positive enhancement of the signal [61]. We expressed ME as a percentage, where 100% means no matrix effect and values above or below indicate positive or negative ME, respectively. Two analytes exhibited positive ME effect, the strongest values were observed for salicylic acid (133.62%), followed by abscisic acid (106.27%). Almost minimal ME was observed also for jasmonic acid (96.72%). We could therefore conclude very little or positive ME for analytes monitored in negative mode. For compounds analysed in positive mode, IAA-Glu, IAA and iPR were the least affected. ACC has the most pronounced negative ME (23.74%), despite high recovery (88.17%). ME and RE are intertwined with the last examined validation parameter, process efficiency. PE values ranged from 17.69% for JA-Ile and 21.01% for ACC up to 106.88% for SA. The PE results are clearly very influenced by ME. Since our main goal was to provide a validated profiling method for ACC, we examined also ACC peaks of synthetic standards, plant extracts and spiked plant extracts by comparison of their retention time and possible peak shape irregularities. The representative LC-MS/MS chromatograms are displayed in Fig. 2. Further, we proceeded to establish a reproducibility test of the LC-MS/MS method by separately injecting three different concentrations of standard (each containing a constant concentration of IS) mixtures (See “Method validation”). The time between each following measurements is approximately 48 h, the solutions were kept in autosampler at 6 °C. The results of this inter-day stability show very good reproducibility for low and medium concentrations, with a bit higher differences for ABA and JA at high concentrations, nevertheless, they always stayed below 15%. The results, with a description of examined standards amount, are shown in Table 3. Autosampler stability was tested for plant extracts containing only internal standards by comparison of the analyte/IS values (Additional file 1: Table S1). The autosampler was again kept at 6 °C and the samples were measured 72 h apart.

**Table 3:** LC-MS/MS method reproducibility test

Analyte	Reproducibility (*n* = 3, %RSD)		
	Low	Medium	High
ACC	0.19	0.12	0.75
*t*Z	0.92	1.31	8.05
*c*Z	1.09	0.59	8.75
iP	1.45	2.99	5.84
*t*ZR	4.00	2.02	7.67
*c*ZR	0.66	5.21	7.92
iPR	1.70	1.48	10.03
IAA	1.28	1.09	6.81
oxIAA	1.21	2.74	8.44
IAA-Glu	0.56	0.47	10.23
IAA-Asp	2.05	2.09	9.19
ABA	4.25	5.89	14.69
JA	0.58	2.89	13.18
JA-Ile	5.66	2.00	5.78
SA	4.40	2.35	8.41

The reproducibility test was performed by injecting three different concentrations (low, medium and high) of standard mixtures in three days from the same sample vial. The amounts (pmol per injection) for each compound (low; medium; high) were as following: ACC, JA-Ile (0.045; 0.45; 4.5); *t*Z, *c*Z, iP, *t*ZR, *c*ZR, iPR (0.005; 0.05; 0.5); IAA, oxIAA, IAA-Glu, IAA-Asp, ABA (0.45; 4.5; 45); JA and SA (0.225; 2.25; 22.5). Results in %RSD difference between normalized peak area, *n* = 3

### Profiling of Arabidopsis mutant plants

After successful method validation, we examined the quantities of analytes in *A. thaliana* and its selected mutants. The compounds were measured in whole plants, shoots and roots from four replicates. The resulting levels in the whole plant are displayed in Table 4 and the levels for roots and shoots in Additional file 1: Table S2.

**Table 4:** Analyte levels in whole seedlings of *A. thaliana* Col-0 and *eto2*, *ein2-1* ethylene mutant lines

Analyte	Col-0	*ein2-1*	*eto2*
ACC	611.8 ± 86.0	4512.1 ± 1056.9	2064.0 ± 864.1
*t*Z	0.42 ± 0.07	0.39 ± 0.03	0.31 ± 0.01
*c*Z	0.57 ± 0.15	0.49 ± 0.09	0.68 ± 0.07
iP	0.40 ± 0.04	0.41 ± 0.04	0.65 ± 0.05
*t*ZR	0.82 ± 0.08	0.83 ± 0.05	1.11 ± 0.14
*c*ZR	2.66 ± 0.81	0.18 ± 0.05	0.17 ± 0.02
iPR	0.74 ± 0.15	0.35 ± 0.09	0.25 ± 0.02
IAA	144.1 ± 7.2	111.3 ± 12.4	133.9 ± 4.5
oxIAA	792.9 ± 67.9	399.0 ± 79.9	285.2 ± 30.0
IAA-Glu	12.67 ± 3.84	33.43 ± 9.42	111.25 ± 22.72
IAA-Asp	50.49 ± 4.83	41.61 ± 4.64	47.06 ± 7.29
ABA	29.25 ± 2.08	25.43 ± 5.44	10.83 ± 1.71
JA	23.00 ± 7.89	41.21 ± 9.06	5.85 ± 1.95*
JA-Ile	15.47 ± 6.87*	7.38 ± 3.12*	ND
SA	349.55 ± 21.94	568.62 ± 80.83	338.67 ± 31.98

Results expressed in pmol/g FW (fresh weight) as means ± SD, *n* = 4, except * where *n* = 3. ND – not detected

Col-0 represents wild type *A. thaliana, eto2* is an ethylene overproducing mutant [62] and *ein2-1* stands for ethylene-insensitive signaling mutant [63]. Phytohormone and related compounds levels in Col-0 were generally in the range determined by other methods – for CK [46, 52, 64]; AUX [48, 49, 52]; JA, JA-Ile, ABA and SA [47, 52]. ACC levels were determined before in different tissues or at different plant ages, they are therefore harder to compare. Many other specific factors could also quickly affect ethylene - ACC levels, such as small changes in temperature [65], circadian rhythms [66], light intensity [67] and others [68]. Nevertheless, our obtained values for FW samples (~ 600 pmol/g for whole plant and root, ~ 1000 pmol/g in shoot) align well with previously reported numbers. Some other results obtained from FW samples are - Bulens et al. [20] reported ~ 50 pmol/g and 2550 pmol/g for unripe and ripe tomato fruit, Sun et al. [69] ~ 1600 pmol/g for *A. thaliana* seedlings, Schellingen e*t al.* ~100 pmol/g for roots and leaves of *A. thaliana* [70], Ziegler et al. measured 320 pmol/g in *A. thaliana* leaves [27]. For whole plants, our measured levels of ACC in *eto2* are elevated compared to Col-0, which was expected, as it is a known ethylene overproducer [71]. Arabidopsis *ein2-1* mutant plants are also known to synthesize significantly more ethylene than wild type [72] and the highest levels of ACC in whole plants were measured there. The highest ACC levels overall were in the roots of the *eto2* mutant. These effects, in these two mutants, could be explained by a potentially disrupted link in biosynthesis feedback loop regulation. Ethylene – ACC metabolism is directly influenced by other phytohormones and vice-versa [3, 5, 73], with IAA interplay shown as probably the most important [33, 74]. In our measurements, there are indeed elevated IAA levels in the roots of all mutants, co-aligning with higher ACC levels there, however this trend was not observed for shoots. A similar pattern is present for salicylic acid, although SA is generally believed to suppress ethylene production or signaling [75]. CKs have been shown to interact with ethylene in the sense of upregulating ethylene biosynthesis [76]. Our results show, that CK levels, except for ribosides in *ein2-1* roots, generally stay the same. ABA, JA and JA-Ile levels are also known to be affected by ethylene-ACC levels [16]. For JA we detect overall lower levels in mutants, with exception of *ein2-1* whole plants. JA-Ile content was not detected for some parts, and it was mostly lower than in Col-0, which aligns with JA levels. ABA levels were lower in the *eto2* mutant.

## Conclusion

Upon discovery of the possibility to measure ACC by a simple derivatization method in plant material, we set forth and tried to include also other phytohormones in quantification. Our main goal was then to test and validate the simplest possible plant extraction method, which would enable us to unequivocally profile important phytohormones from *A. thaliana* tissues, as these molecules and plant material represent one of the main instruments of plant science. This method was analytically examined and proved to be applicable, resulting in one of the first validated method employing internal standard for exact 1-aminocyclopropane-1-carboxylic acid profiling in *A. thaliana*.

## Methods

### Chemicals

Cytokinin labeled and nonlabeled standards (see Table 1), [^2^H_6_]-ABA; JA; [^2^H_6_]-JA; JA-Ile; [^2^H_2_]-(-)-JA-Ile; oxIAA; IAA-Asp; IAA-Glu; [^13^C_6_]-IAA; [^13^C_6_]-IAA-Asp and [^13^C_6_]-IAA-Glu were obtained from Olchemim Ltd. (Olomouc, Czech republic). [^13^C_6_]-oxIAA came from laboratory library of standards [47]. ACC; [^2^H_4_]-ACC; SA; [^2^H_4_]-SA; ABA and IAA were acquired from Sigma Aldrich (St. Louis, MO, USA). AccQ-Tag™ Ultra Derivatization Kit for UPLC Amino Acid Analysis is from Waters Inc. (Milford, MA, USA). All other chemicals were of analytical or higher grade from Sigma Aldrich (St. Louis, MO, USA).

### Plant material

The mutant plants used in this study, *eto2* [62] and *ein2-1* [63] were previously described. *Arabidopsis thaliana* (L.) Columbia-0 (Col-0) seeds, and mutant plant seeds were first sterilized using a specific solution (70% ethanol solution supplemented with 0.1% TWEEN-20) for 10 min, followed by washing with sterilized dH_2_O water. They were stratified for 3 days in 4 °C and sown on half-strength solid Murashige and Skoog medium supplemented with 1% sucrose (0.22% MS media, 1% agar, pH 5.7) in square Petri dishes, then placed in growth chamber at 21 ± 1 °C, under long-day photoperiod (16 h light, 8 h dark) with a light intensity of 110 µmol photons m^− 2^ s^− 1^. 10 days after germination, the samples (whole plants, roots and shoots) were collected, snap frozen with liquid nitrogen and stored in -80 °C for further use in all of the experiments.

### Extraction of plant samples

10 DAG old *A. thaliana* were snap frozen after collection, homogenized in liquid nitrogen and reweighed into ~ 10 mg aliquots for all validation and method development experiments. For profiling of Arabidopsis mutant plants, the samples were weighed independently, without homogenization, as biological replicates. Extraction was performed by adding 500 µL of cold 10% ACN with 0.5% formic acid (v/v), with or without internal standards cocktail dissolved in dH_2_O, to the plant material. Also, three zirconium oxide 2 mm beads, ceria-stabilized (Next Advance) were added and the resulting mixture was homogenized using 2 × 3 min runs at 30 Hz in pre-cooled clamps on a bead mill (Retsch GmbH, Haan, Germany). The samples were then centrifuged at 30 000 g and 4 °C for 15 min. Afterwards, the supernatant from one sample was collected, 150 µL was used for ACC quantification and 250 µL for the remaining phytohormones and their metabolites profiling. The supernatants were evaporated to dryness on rotary vacuum evaporator. The samples were then adjusted for LC-MS/MS analysis - for phytohormones dissolved in 30 µL of 10% ACN (v/v) and for ACC derivatized by adding 14 µL of borate buffer and 4 µL of derivatization reagent, prepared according to manufacturer’s instructions. A detailed schematics of sample extraction is showed in Additional file 1: Figure S2.

### LC-MS/MS conditions and instrumentation

Quantification measurements were performed on Agilent 6495B Triple-Quad LC/MS coupled to 1260 Infinity II LC system (Agilent Technologies, Inc., Santa Clara, CA, USA). The resulting data were quantified and processed using MassHunter Workstation Software ver. B.09.00 (Agilent Technologies). The mass spectrometer was operated in “Dynamic MRM” mode, with following source parameters - gas temp 160 ^o^C, gas flow 14 L/min, sheat gas temp 390 ^o^C and shear gas flow 12 L/min. The capillary voltage was set to 2800 V for positive mode and 3000 V for negative mode, with nozzle voltage at constant 0 V.

The chromatographic columns, Kinetex Biphenyl (100 × 2.1 mm, 1.7 μm) and Kinetex Polar (150 × 2.1 mm, 2.6 μm), were from Phenomenex Ltd. (Phenomenex, Torrance, CA, USA). Kinetex Polar, with inlet filter, was used for all presented work. Gradient employed for all analyses takes 23 min at flow of 0.53 mL/min and consists of mobile phase A (dH_2_O with 0.03% FA (v/v)) and B (acetonitrile). Its gradient time-frame was: 0 min − 99% A, 8 min – 91.5% A, 9 min – 90% A, 15 min – 88% A, 20 min – 2% A, 21 min – 99% A, 23 min – 99% A. The column compartment was heated to 53 ^o^C.

### Method validation

For accurate quantification, calibration curves were prepared by using the listed standards and isotopic standards (Table 1), added in known concentrations. Quantification of the samples was performed using the standard isotope dilution method [77], where a ratio of endogenous or standard compound to labeled internal standard (IS) is multiplied by IS concentration and used to compute the endogenous or standard compound quantity in the sample.

For method validation experiments, three sample groups were prepared in four replicates. For the initial group, 10 mg of *A. thaliana* samples were spiked with known amounts of internal standards at the beginning of extraction. For the second group, IS was added together with varying amounts of spiking solution, containing nonlabeled standards, at the start of extraction. Third group was prepared by adding IS at the beginning of extraction and by spiking the sample with nonlabeled standards of different concentrations, matching the ones from second sample set, before evaporation. Except for adding the standards, all the samples were processed the same way and using the same amount of plant material (see “Extraction of plant samples”). The concentration of targeted nonlabeled analytes introduced to second group of samples were computed by subtraction of their endogenous levels (obtained from first sample group). Subsequently, the obtained results were compared with known theoretical quantity of standards spiked into sample. The results of this comparison are presented as method accuracy, displaying the percentage bias (Table 2). For each analyte, the method precision was then expressed as average RSD (in percent) of analyte quantity obtained from the four replicates of each spiked amount. Analyte recovery from the extraction procedure was determined by dividing the peak area of nonlabeled analyte spiked at the beginning of extraction (sample group 2) to the mean peak area of matching analyte spiked before evaporation (sample group 3), in four replicates. The value is expressed in percentage (Table 2) from all obtained values. Process efficiency and matrix effect were calculated according to [60] and expressed as % of relative standard deviations (RSD).

The autosampler stability was measured in four replicates of extracted plant material containing IS, on two different days. The normalized area of every analyte was calculated (analyte/IS) and the means for day 1 and 2 compared. The result is expressed as %RSD of day 1 and 2 means difference, see Additional file 1: Table S1. Day 1 and 2 measurements were approximately 72 h apart and the samples were kept in autosampler at 6 °C for the whole duration of the experiment.

To assess the reproducibility of the method, mixtures of three different standard concentrations of each compound, containing constant amount of IS, were prepared and injected once on three different days, each concentration from the same sample vial. The difference between day 1 and 2, and day 2 and 3 was approximately 48 h, with samples placed in autosampler at 6 °C for the whole duration. The resulting three values for each examined concentration, calculated as normalized area (analyte/IS), were compared and are displayed in Table 3 as %RSD. The concentrations of nonlabeled standards (low, medium and high) were as follows, in pmol per injection – ACC, JA-Ile (0.045; 0.45; 4.5); *t*Z, *c*Z, iP, *t*ZR, *c*ZR, iPR (0.005; 0.05; 0.5); IAA, oxIAA, IAA-Glu, IAA-Asp, ABA (0.45; 4.5; 45); JA and SA (0.225; 2.25; 22.5).

### Electronic supplementary material

Below is the link to the electronic supplementary material.

Supplementary Material 1

## Data Availability

Data are available upon request from the corresponding author.

## Abbreviations

ABA: abscisic acid
ACC: 1-aminocyclopropane-1-carboxylic acid
ACN: acetonitrile
ACO: ACC oxidase
ACS: ACC synthase
AUX: auxins
CK: cytokinins
*c*Z: *cis*-zeatin
*c*ZR: *cis*-zeatin riboside
DAG: days after germination
dH_2_O: deionized water
ESI: electrospray ionization
FW: fresh weight
GC: gas chromatography
HPLC: high-performance liquid chromatography
IAA: indole-3-acetic acid
IAA-Asp: indole-3-acetyl aspartate
IAA-Glu: indole-3-acetyl glutamate
iP: isopentenyladenine
iPR: isopentenyladenine riboside
IS: internal standard
JA: jasmonic acid
JA-Ile: jasmonyl-isoleucine
LC: liquid chromatography
LC-MS/MS: liquid chromatography coupled with tandem mass spectrometry
LLE: liquid-phase extraction
ME: matrix effect
MRM: multiple reaction monitoring
MTA: 5’-methylthioadenosine
oxIAA: 2-oxindole-3-acetic acid
PE: process efficiency
RE: recovery
RSD: relative standard deviation
SA: salicylic acid
SAM: *S*-adenosyl-l-methionine
SD: standard deviation
SPE: solid-phase extraction
*t*Z: *trans*-zeatin
*t*ZR: *trans*-zeatin riboside
UHPLC: Ultra High Performance Liquid Chromatography
