# Supplementary material for: Profiling of 1-aminocyclopropane-1-carboxylic acid and selected phytohormones in Arabidopsis using liquid chromatography-tandem mass spectrometry
Source: Plant Methods. 2024 Mar 16;20:41. doi: 10.1186/s13007-024-01165-8 (PMC10943774; doi:10.1186/s13007-024-01165-8)
Supplement: Supplementary file 1 — Supplementary Material 1 [file 13007_2024_1165_MOESM1_ESM.pdf]

Additional file 1

Additional file 1: Figure S1. Proposed scheme of 1-aminocyclopropane-1-carboxylic acid (ACC) derivatization with AccQ-Tag^{TM} Ultra Derivatization Kit, with fragmentation

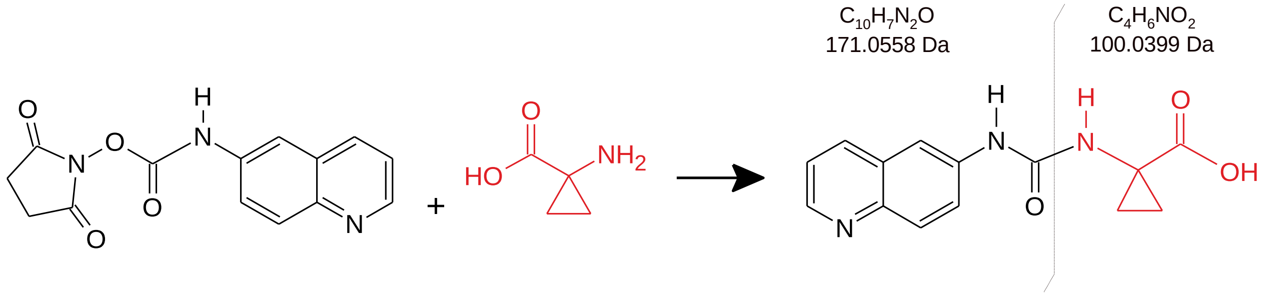

AQC ACC AMQ ACC

The proposed fragmentation is marked with a separating sign, with monoisotopic masses in Daltons (Da) of fragments atom summary formula, corresponding to *m/z* (mass-to-charge ratio) detected in LC-MS/MS.

AQC – 6-aminoquinolyl-*N*-hydroxysuccinimidyl carbamate; AMQ – amino-quinoline moiety

Additional file 1: Figure S2. Sample extraction and analysis workflow schematics

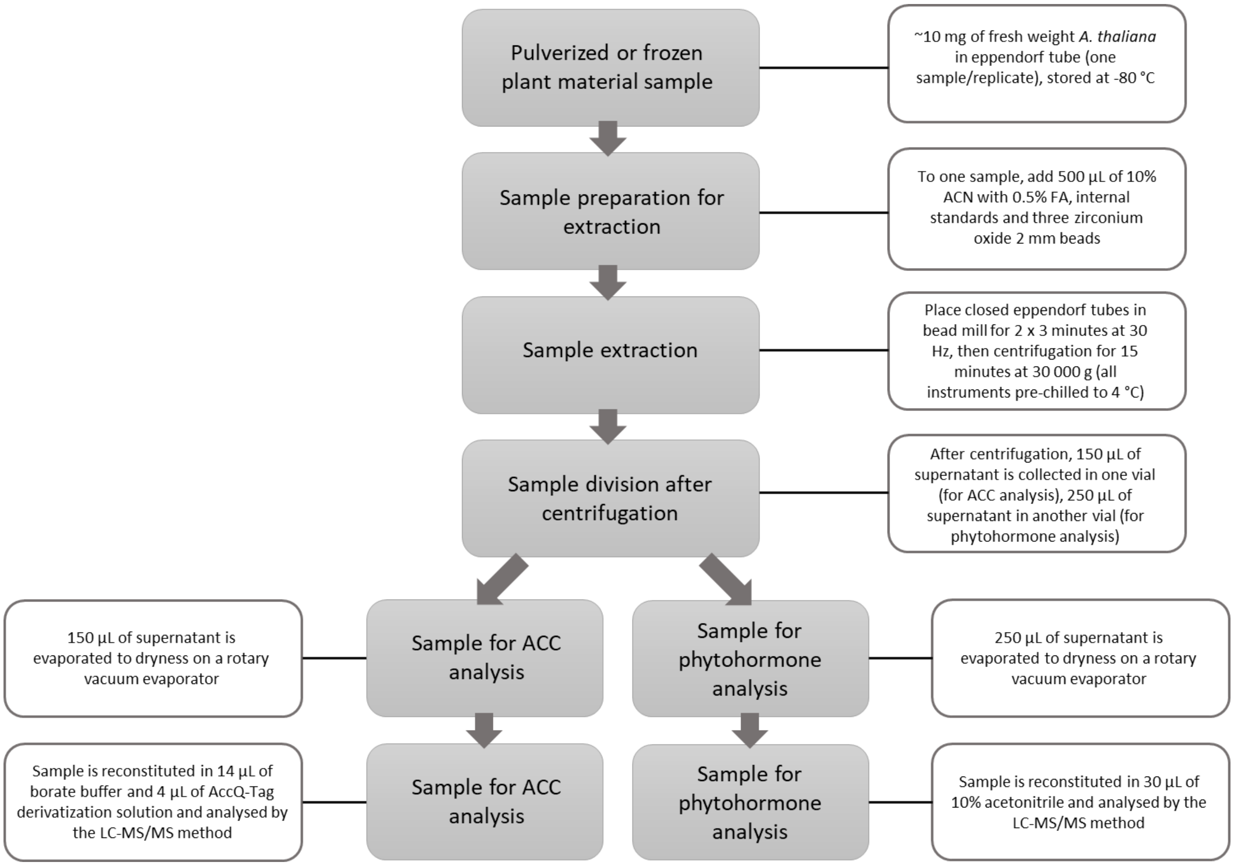

Additional file 1: Table S1. Autosampler stability

Analyte	Autosampler Stability (n=4; %RSD)
**ACC**	0.45
***t*Z**	4.24
***c*Z**	3.92
**iP**	7.50
***t*ZR**	13.02
***c*ZR**	4.77
**iPR**	8.46
**IAA**	3.59
**oxIAA**	6.82
**IAA-Glu**	1.00
**IAA-Asp**	1.34
**ABA**	0.54
**JA**	1.78
**JA-Ile**	5.59
**SA**	5.38

Autosampler stability was examined by analyzing the same four extracted 10 mg samples of *A.thaliana* plants, with added IS, on two separate days. Each measurement result was expressed as normalized peak area (analyte/IS), and the difference of means between the two separate day measurements is expressed as %RSD. Days 1 and 2 were approximately 72 hours apart, with samples kept in the autosampler for the whole experiment duration.

Additional file 1: Table S2. Analyte levels in root and shoot of *A.thaliana* seedlings Col-0 and *eto2*, *ein2-1* ethylene mutant

Analyte	Col-0						*ein2-1*						*eto2*					
	Root			Shoot			Root			Shoot			Root			Shoot		
**ACC**	623.0	±	125.4	1046.9	±	197.8	4376.2	±	715.5	3674.3	±	1494.1	7139.4	±	1638.5	2303.2	±	199.6
***t*Z**	1.17	±	0.23	0.29	±	0.02	1.57	±	0.33	0.21	±	0.01	0.64	±	0.07	0.24	±	0.03
***c*Z**	2.73	±	0.35	0.44	±	0.02	2.63	±	0.34	0.23	±	0.03	2.62	±	0.16	0.35	±	0.03
**iP**	1.07	±	0.07	0.13	±	0.09	1.51	±	0.13	0.11	±	0.02	2.18	±	0.24	0.28	±	0.04
***t*ZR**	2.39	±	0.34	0.65	±	0.10	28.29	±	10.54	0.50	±	0.07	2.71	±	0.34	0.70	±	0.04
***c*ZR**	6.55	±	1.22	0.67	±	0.04	5.64	±	2.71	0.26	±	0.03	0.65	±	0.04	0.25	±	0.04
**iPR**	1.64	±	0.36	0.32	±	0.06	2.92	±	1.13	0.28	±	0.03	0.64	±	0.01	0.28	±	0.05
**IAA**	292.7	±	14.2	135.4	±	2.4	543.5	±	112.9	135.0	±	9.2	409.4	±	107.0	112.9	±	13.2
**oxIAA**	2158.0	±	160.0	2529.6	±	82.3	1537.4	±	361.9	592.3	±	123.4	649.5	±	24.5	601.5	±	163.5
**IAA-Glu**	19.49	±	5.75	9.29	±	4.87	138.81	±	15.66	4.37	±	0.97	77.65	±	11.84	30.53	±	14.43
**IAA-Asp**	89.86	±	11.73	34.32	±	3.03	33.81	±	14.77	46.56	±	4.87	53.11	±	7.75	63.19	±	20.31
**ABA**	19.99	±	1.99	7.11	±	1.99	35.23	±	5.58	29.14	±	4.14	7.18	±	1.07	9.38	±	2.77
**JA**	48.19	±	12.89	122.59	±	29.94	35.14	±	19.82	36.70	±	9.05	13.83	±	6.88	31.89	±	13.25
**JA-Ile***	51.38	±	16.41	218.40	±	58.15	ND			60.83	±	2.89	ND			34.70	±	12.71
**SA**	591.24	±	184.19	214.00	±	14.59	2524.85	±	208.61	544.09	±	33.27	749.80	±	137.24	217.35	±	52.71

Results expressed in pmol/g FW (fresh weight) as means ± SD, n=4, except JA-Ile*, where values (n=3).

ND – not detected.
